# Fatal intoxication after oral ingestion of amphetamine: Two case reports

**DOI:** 10.1016/j.fsisyn.2024.100568

**Published:** 2025-01-28

**Authors:** Evelyn Pawlik, Felix Mayer, Oliver Temme

**Affiliations:** a Forensic Toxicology, Institute of Legal Medicine Duesseldorf, Moorenstr. 5, 40225, Duesseldorf, NRW, Germany; b Section, Institute of Legal Medicine Duesseldorf, Moorenstr. 5, 40225, Duesseldorf, NRW, Germany

**Keywords:** Amphetamine, Leuckart synthesis, 4-M-5-PP, DPIA, Distribution

## Abstract

Amphetamine is a stimulant that is abused worldwide and e.g. leads to hyperthermia [Brinkman et al., 2014], dizziness, insomnia, stomachaches and suppression of appetite [Callaway et al., 1994]. The most common production route of racemic (R-/S)-amphetamine is the Leuckart synthesis [United Nations Office on Drugs and Crime 2006, Hauser et al., 2018], where by-products like 4-methyl-5-phenylpyrimidine (4-M-5-PP), N,N-di (β-phenylisopropyl)amine (DPIA) and N-formylamphetamine (NFA) are incurred. We describe two cases in which 39 years old men died after oral intake of greater amounts of liquid amphetamine preparations. Body fluids (heart blood, femoral vein blood, urine, cerebrospinal fluid, vitreous humour, and stomach content), organ tissues (myocardium, lung, liver, gall bladder, brain and kidney) and skeletal muscle were examined for amphetamine and amphetamine by-products as well as for other substances e.g. alcohol and pharmaceuticals. Analysis were done via HPLC/DAD, LC/MS, GC/MS or GC/FID without or after fluid-fluid extraction. Amphetamine was detected in all biological materials, the highest concentrations were found in urine (2600 μg/ml, case 1) and stomach content (14,000 μg/g, case 2). The amphetamine by-product DPIA was found only in heart blood (case 2), while NFA and 4-M-5-PP could not be detected at all. Morphological findings and the toxicological results for (R-/S)- amphetamine, the amphetamine by-products, alcohol, other drugs and pharmaceuticals are shown for both cases. The amphetamine concentrations of both cases are compared and the distribution in the body is discussed. The toxicity of the amphetamine by-products on the human body remains unclear and is subject of further studies.

## 1 Introduction

In 2021, 36 million people abused the stimulant amphetamine worldwide which is structurally related to phenylethylamine [25]. Stimulant drugs like cocaine, 3,4-methyldioxy-N-methamphetamine (MDMA), ephedrine and fenethylline (a theophylline derivate of amphetamine) are frequently found in forensic cases [32], though differences in drug abuse varies between countries. People in drug treatment in European countries and most subregions of Asia have stated, that they abuse opioids, in Latin America cocaine, in parts of Africa (predominantly in south and central Africa) cannabis and in east and south-east Asia methamphetamine as the primary drug [25]. Fenethylline, sold as tablets under several trade names like Fitton and Biocapton but best known as Captagon®, is traditionally consumed on the Arabian Peninsula and since 2013 also in North Africa [29]. Today the illicit drug is just called “captagon” after the original trade name [30]. Clandestine laboratories producing fenethylline are found to be located in Bulgaria, Slovenia, Serbia and Montenegro [29]. Germany, Belgium, France and the Netherlands allowed the legal sale of Captagon® until 2013 for the treatment of narcolepsy, with illegally trafficked pills subject to seizure [30]. The German Federal Office of Criminal Investigation (BKA) registered 284.603 infringements against the narcotic law in 2019 of which 39.607 were related to amphetamine with rising numbers until 2020 [16] and a decrease in the following years until 2023 [24]. Amphetamine and amphetamine derivates are the most commonly used stimulant in Europe, however, other stimulants like synthetic cathinones, methamphetamine or new psychoactive substances are becoming more popular in Europe [23,28].

In both clandestine and pharmaceutical laboratories, the Leuckart reaction provides a simple synthetic route for the synthesis of racemic amphetamine [3,4]. Leuckart discovered this method in 1885 while researching the reaction of benzaldehyde with formamide to benzylidenediformamide [5]. Besides amphetamine, other by-products such as 4-methyl-5-phenylpyrimidine (4-M-5-PP), N, N-di (β-phenylisopropyl)amine (DPIA) and N-formylamphetamine (NFA) [4] can be formed during the synthesis. These by-products can originate from impurities already present in the starting material, from compounds which are formed from impurities in the starting material, from an incomplete reaction of the starting material or amphetamine intermediates or an inadequate purification of the intermediates formed during the synthesis [31]. Amphetamine is composed of two active forms: the levoamphetamine (R)- and the dextroamphetamine (S)-enantiomer. Dextroamphetamine is 3–4 times more potent than levoamphetamine [6] and is metabolized to a higher rate than the (R)-form [7]. Due to its structural similarity to the endogenous catecholamine neurotransmitters noradrenaline and dopamine, amphetamine acts as a competitive substrate for the monoamine reuptake transporters, releases noradrenalin and dopamine from the vesicles into the cytosol of the presynaptic cell and inhibits the monoamine oxidase (MAO) [8]. These processes are the reasons for the most common, dose-dependent side effects of amphetamine including the influence on the cardiovascular as well as on the gastrointestinal system [9,10] and its recreational abuse. Amphetamine suppresses appetite and fatigue, it also causes dizziness, insomnia, stomachaches, nausea, vomiting, dry mouth, arousal and anxiety, aggressiveness, hyperactivity, euphoria, intensified emotions, boosted self-esteem and hyperthermia [1,2,11]. In case of an overdose, it leads to tachycardia, fever, respiratory depression, convulsions, coma, circulatory collapse and even death [1,[11], [12], [13],^26^]. After death post-mortem redistribution processes may occur, which can lead to misinterpretation of the concentrations found in blood samples. Therefor brain tissue and cerebrospinal fluid can offer a clearer picture since they are less affected by post-mortem redistribution [14]. For amphetamine it was shown that the drug undergoes only slightly redistributed in the body after death [21]. This article intents to discuss the concentration differences in body fluids and tissues as well as the impact of detected by-products with regard to the cause of death on the basis of two case studies, as there are several problems hindering the evaluation of the cause of death in fatalities related to the abuse of amphetamine.

## 2 Case presentation

### 2.1 Report 1

A 39-year-old man with a long history of excessive drug consumption, including alcohol, ecstasy and LSD got a visit from his friend, who noticed that he was obviously "high". The man appeared to be drunk, had a slurred pronunciation and was sweating. He admitted feeling indisposed and wanted to lie down for a moment. His friend helped him to bed, made him a calf packing and offered him water, sugar water and milk. The man stated that he had drunk some amphetamine and would soon be on track again. 30–45 minutes later his friend checked on him and found that he had respiratory problems (he gasped) and was no longer responsive. The consulted emergency physician cancelled the unsuccessful resuscitation measures. Inside the man's apartment the police found a plastic box labeled "Vasopro Ephedrin" an empty plastic box with a painted skull and paraphernalia for consuming narcotic drugs (a glass plate with white powder adhesions, a card, and a self-made snorting tube), as well as pressure strip bags with powder adhesions and ecstasy tablets. In addition, they found alcohol and various partly unused drug blisters (diazepam, doxepin, paracetamol and methylphenidate), pipamperone and promethazine. His bed showed stains of vomit.

### 2.2 Report 2

A 39-year-old man drove together with his girlfriend to a friend's apartment to pick up some things. He went into the kitchen and drank an unknown liquid from a bottle. Afterwards he complained about pain and a burning sensation in the mouth and throat, wherefore he rinsed his mouth. He told his girlfriend that he was feeling sick and that his mouth was feeling numb. He was pale, his eyes were wide, his breath smelled “chemically” and he was sweating strongly. His girlfriend offered him a glass of water and salt to force him to vomit. Shortly after he had vomited something “bright yellow”, he started to cramp. His girlfriend called friends who helped her to cool him down with wet towels. He was no longer able to speak, his mouth became very narrow, and saliva ran out it. When the ambulance arrived, he appeared blue, his eyes were closed, he had bitten his tongue and was already asystolic. During the cardiopulmonary resuscitation the emergency physician found injuries in his mouth and throat, indicating a chemical burn, and noticed that his vomit smelled of lye.

## 3 Materials and methods

### 3.1 Autopsy and histopathological examinations

Autopsies were performed five days (case 1) and one day (case 2) after death according to the guidelines of the Working Group of the Scientific Medical Societies in Germany (Die rechtsmedizinische Leichenöffnung [15]) including the preservation of a standard set of samples of body fluids and tissues for toxicological analysis. Histological examinations comprised the preparation of fine tissue sections from formalin-fixed and paraffin-embedded tissue samples which were stained with standard staining methods (haematoxylin-eosin, Elastica-van-Gieson, periodic-acid-Schiff, Prussian-blue).

### 3.2 Toxicological analysis

Toxicological analysis on the biological specimens (taken during autopsy) were performed in accordance to the society for toxicological and forensic chemistry (GTFCh). Heart blood was mixed with 10 mg/ml sodium fluoride before storing with all other specimens at −20 °C. Preliminary screening of heart blood and urine as well as general unknown screenings were done routinely by immunoassay technique (Indiko, Thermo Fisher), via HPLC/DAD (Waters W1515, Photodiode Array Detector 2996 (PDA)) and LC/MS (Waters Aquity) after liquid-liquid extraction. The blood ethanol concentration was determined by enzymatic ADH method (Pentra C400, Fa. Horiba Medical) and headspace-GC coupled with a flame ionization detector (HS 110/Clarus 500, Fa. PerkinElmer). The quantification of amphetamine in all analysed fluids and tissues were performed with GC/MS. Appropriate dilutions of body fluids or tissues were mixed with 50 μl of the internal standard (amphetamine-d11, Fa. Cerilliant) and 50 μl 2 M sodium hydroxide (Fa. Merck, VWR) and extracted with 500 μl isooctane (suprasolv Fa. Merck). 200 μl of the supernatant obtained after liquid-liquid extraction were mixed with the derivatization reagent (N-methyl-bis-trifluoroacetamide (MBTFA), Machery-Nagel) and incubated in an oven for 30 minutes at 90 °C. The analysis were performed on an Agilent 6890N gas chromatograph with an Agilent 5975i mass spectrometer and the MSD-chemstation software E 02.00 under the following conditions: HP-5 MS (5 % phenyl)methylpolysiloxan) capillary column from Agilent (length: 30 m, inner diameter: 0.25 mm, film thickness: 0.25 μm), carrier gas: helium 5.0 in SIM mode. Determination of the (S)- and (R)-enan tiomer were performed on an GC/MS-System (Agilent) with a chiral cyclodextrine column (MEGA DEX-G01) from Fa. MEGA (length: 30 m, inner diameter: 0.25 mm, film thickness: 0.25 μm), carrier gas: helium 5.0 in SIM mode. Amphetamine by-products were liquid-liquid-extracted with chlorobutane, derivatized with MBTFA and analysed on the same GC/MS system mentioned above.

## 4 Results

### 4.1 Report 1

#### 4.1.1 Autopsy findings

Lungs were voluminous and rich with blood, they presented a pronounced oedema and signs of vomit aspiration (chyme in the mouth, nose and respiratory tract; faveolate discoloration of lung tissue). The heart was enlarged with an increase in muscle mass and coronary arteries presented low-grade atherosclerosis. A fracture of the sternum was related to cardio-pulmonary resuscitation.

#### 4.1.2 Histopathological findings

Tissues sections of myocardium revealed hypertrophy-related alterations of myocardiocytes, several smalls fibrous scars and a nearly healed myocardial infarction with discreet remains of granulation tissue in the rear wall of the left ventricle. Lung tissue samples showed discreet anthracosis, a predominantly subpleural located emphysema, sparse signs of chronic inflammations, discreet intra-alveolar oedema and chronic congestion. Liver tissue presented a beginning fatty degeneration.

#### 4.1.3 Toxicological findings

The toxicological examination of femoral vein blood did not detect any ethanol. The immunochemical heart blood and urine analysis were positive for amphetamine and its analogues. General unknown screening via HPLC/DAD and LC/MS was positive for pipamperone, doxepine, diazepam and its metabolites (nordazepam, temazepam), trimipramine and methylphenidate. In all samples high concentrations of amphetamine (racemic) could be detected, since the highest concentration was found in the urine and the stomach. The results of all toxicological analysis (organs and body fluids) as well as the concentrations for amphetamine ((S)- and (R)-enantiomer) are shown in Table 1.

**Table 1:** Concentrations of amphetamine and all routinely examined substances (other drugs and pharmaceuticals) in organ tissues (myocardium, lung, liver, gall bladder, brain, kidney) as well as in skeletal muscle in [μg/g] and body fluids (heart blood, femoral vein blood, urine, cerebrospinal fluid, vitreous humour, stomach content) in [μg/ml] for case 1 and case 2. The amphetamine by-products DPIA, NFA and 4-M-5-PP as well as some other pharmaceuticals were just analysed qualitative.

Biological material	Concentration [μg/ml] and [μg/g] respectively			
	amphetamine		other substances	
	Case 1	Case 2	Case 1	Case 2
Heart blood	73.7 (racemic) (R-) 36.9 (S-) 36.2	255 (racemic) (R-) 143 (S-) 135	pipamperone approx. 0.250, doxepin n. d., diazepam 0.029, nordazepam 0.005, DPIA, NFA, 4-M-5-PP n. d.	DPIA p. n. q., NFA, 4-M-5-PP n. d.
Femoral vein blood	41.8	72.1	DPIA, NFA, 4-M-5-PP n. d.	DPIA, NFA, 4-M-5-PP n. d.
Urine	2600	141	pipamperone p. n. q., doxepin 0,33, nordazepam and temazepam p. n. q., DPIA, NFA, 4-M-5-PP n. d.	cocaine and its metabolites (benzoylecgonine, ecgonine methyl ester) p. n. q., paracetamol p. n. q., DPIA, NFA, 4-M-5-PP n. d.
Cerebrospinal fluid	43.7	59.5	no more analysis done	no more analysis done
Vitreous humour	37.6	136	no more analysis done	no more analysis done
Myocardium	89.8	92.3	no more analysis done	no more analysis done
Skeletal muscle	68.9	92	no more analysis done	no more analysis done
Lung	343	227	no more analysis done	no more analysis done
Liver	229	186	no more analysis done	no more analysis done
Gall bladder	136	590	no more analysis done	no more analysis done
Brain	175	247	doxepin 0.150, trimipramine approx. 0.130	no more analysis done
Kidney	128	95.1	methylphenidate	no more analysis done
Stomach content	1200	14,000	no more analysis done	no more analysis done

n. d.: not detected, p. n. q.: positive not quantitated, DPIA: N,N-di(β-phenylisopropyl)amine, NFA: N-formylamphetamine, 4-M-5-PP: 4-methyl-5-phenylpyrimidine.

### 4.2 Report 2

#### 4.2.1 Autopsy findings

**Fig. 1:**
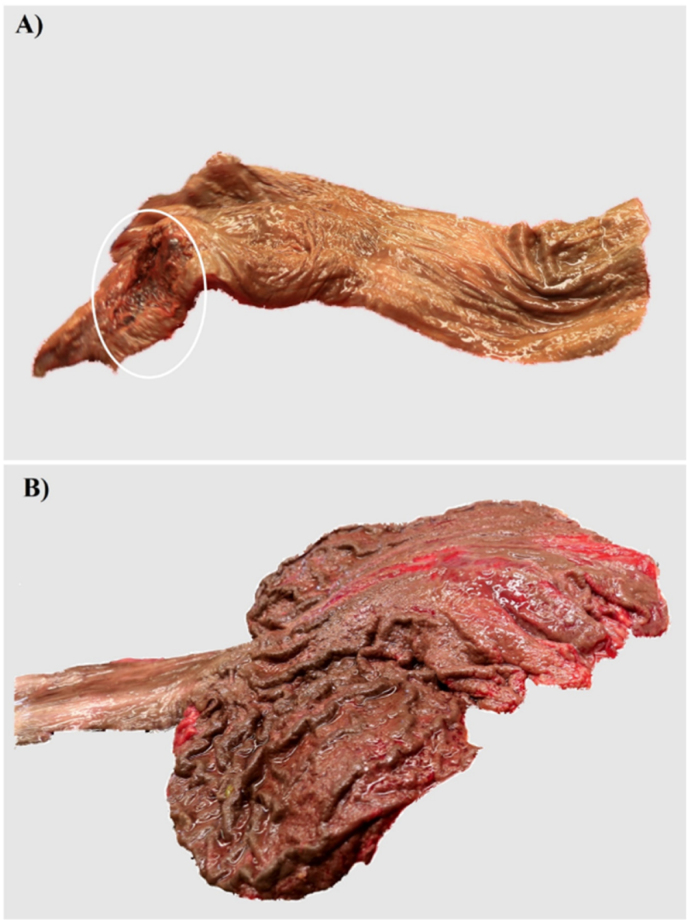
Autopsy findings in case 2 A) Oesophagus with brown discoloration of the mucosa and an extensive tissue injury of the adjacent section of the throat (circle) B) Stomach with brown discoloration of the mucosa and numerous, partly haemorrhagic muscosal ersoions.

The skin around the mouth and the mucous membranes of the oral cavity, throat, oesophagus and stomach showed a striking brown discoloration. There were also extensive injuries of the mucous membranes of the lips, throat and stomach, partly with haemorrhage (Fig. 1). The heart presented moderate increase in myocardial mass, moderate (coronary) arteriosclerosis. Further organ findings were a beginning fatty degeneration of the liver, pulmonary oedema, food particles in the upper respiratory tract and a kidney cyst. The skin showed reddened, superficial injuries in the facial area, a rash of the back and shoulders and scars on the left arm with morphological characteristics implying self-infliction.

#### 4.2.2 Histopathological findings

Samples of soft tissue of the throat revealed coagulation necrosis of mucous membrane and adipose tissue. The myocardium presented perivascular fibrosis and a fibrous scar in the interventricular septum. Lung tissue showed signs of (predominantly subpleural) emphysema, signs of chronic inflammations and anthracosis. Furthermore a fatty degeneration of liver tissue, haemorrhage in the spleen and psoriasis (skin on the back and shoulders) were found.

#### 4.2.3 Toxicological findings

In femoral vein blood an ethanol concentration of 1.13 ‰ was detected. The immunochemical heart blood and urine analysis were positive for amphetamine and its analogues. General unknown screening via HPLC/DAD and LC/MS was positive for paracetamol as well as cocaine and its metabolites respectively. In all samples high concentrations of amphetamine (racemic) could be detected. The highest concentration was found in the gall bladder and the stomach respectively. The results of all toxicological analysis (organs and body fluids) as well as the concentrations for amphetamine ((S)- and (R)-enantiomer) are shown in Table 1.

## 5 Discussion

### 5.1 Cause of death and additional findings

Overdoses with amphetamine are not uncommon but only few cases with such high concentrations as the ones presented here are described [20]. Although fatal amphetamine intoxication with blood concentration less than 0.3 μg/ml may occur, concentrations of 1 μg/ml or higher are not unusual in severe addicts with a high tolerance [18,27]. The concentrations that were detected in the presented cases explain the death of the men without any doubt. Several side effects of amphetamine are reported in cases of amphetamine overdoses. Tachycardia, fever, respiratory depression, convulsions, circulatory collapse and death may occur. In the presented cases, some of the typical side effects were described by witnesses (e.g. sweating, respiratory problems, case 1; cramping, asystolia, case 2). While the morphologic findings (autopsy, histologic examinations) are rather unspecific in case 1, case 2 presents extensive tissue alterations caused by the exposure to acid or lye: the brown discoloration of skin and mucous membranes, the coagulation necrosis of mucous membranes and adipose tissue of the throat as well as the superficial skin injuries of the face are easily explainable by contact with concentrated amphetamine base. Though not relevant for the occurrence of death – there were no organ perforations or signs of extensive bleeding – these findings provide first hints to the chemical characteristics of the consumed liquid. The other drugs detected in these cases, besides amphetamine, are likely to play only a subordinate role, also if interactions e.g. with other simulant drugs like cocaine, which was only found in urine of case 2, are well known.

### 5.2 By-products of amphetamine synthesis

Amphetamine can be snorted, ingested orally or, less common, be injected. The men in both presented cases died shortly after oral intake of greater amounts of liquid amphetamine preparations**.** Forensic analysis findings of both men were compared. In the heart blood, of both cases the (S)- and (R)-enantiomers of amphetamine were separated, and heart blood, femoral vein blood and urine were also analysed for the amphetamine synthesis by-products. The ratio of amphetamine enantiomers in the heart blood were close to 1, showing an ingestion of racemic amphetamine shortly before death, as the (S)-enantiomer is metabolized faster than the (R)-enantiomer [5]. DPIA was the only amphetamine synthesis by-product which could be found in the heart blood (case 2), showing that the ingested amphetamine in this case was synthesized by Leuckart reaction. This suggests that the amphetamine preparation in case 1 was produced by a different synthetic route, the starting materials were completely pure, the amphetamine synthesis was complete with no side reactions or the purification of synthesis intermediates respectively the final product was correct [31]. The same reasons may account for the fact that in both cases 4-M-5-PP and NFA were neither detected in heart blood, nor in femoral vein blood or in urine. It also remains unclear how toxic these by-products are to the human body. This question should be answered through further studies.

### 5.3 Distribution of amphetamine in the body

In the literature, some fatal cases involving amphetamine were described. Meyer et al. [17] reported one case of fatal amphetamine poisoning where a 22 year old male died after consuming this drug. In his blood 2.4 μg/ml and in his brain 5.5 μg/g amphetamine was found. Verschraagen et al. [18] described 70 cases from which 38 cases comprised an amphetamine related cause of death. In seven cases the victims died from the direct effect of amphetamine. The blood concentrations in these cases ranged from 0.24 to 11.3 μg/ml. The amphetamine blood concentrations observed in the present study were 6–30 times higher (case 1) than reported by the other authors, similar to the determined brain concentrations. Orrenius et al. [19] reported 3 cases in which the measured amphetamine blood concentrations (500 ng/ml to 7 μg/ml) as well as the concentrations in liver (12–45 μg/ml) and kidney (4–8 μg/ml) were even many times lower than presented in this study. In addition to the already mentioned studies, Musshoff et al. [20] listed 5 further cases in which the amphetamine concentration was fatal. The blood concentrations ranged from 1.8 to 41 μg/ml (femoral vein blood, heart blood respectively or just unknown), liver concentrations from 11.7 to 45 μg/g and kidney concentrations from 4 to 48 μg/g. The routes of ingestion were oral or intravenous. All these concentrations were much lower than those listed in Table 1, showing that amphetamine has a wide range of fatal concentrations, which were also observed in different biological material. The cases in this study show different amphetamine distribution and post-mortem redistribution patterns in the examined biological materials. Drummer [21] assumes that amphetamine is subject to post-mortem redistribution only to a small extend. Another study shows that amphetamine concentrations decrease after death [22]. In our case 1, the concentration of amphetamine in the lung (343 μg/g) was much higher than in the heart blood (73 μg/ml), showing that the man survived the amphetamine intake at least slightly longer than the man in case 2. In case 2 lung (227 μg/g) and heart blood (255 μg/ml) amphetamine concentrations were nearly the same suggesting that death occurred quite fast. The lowest concentrations of amphetamine were found in the cerebrospinal fluid (43.7 μg/ml (case 1), 59.5 μg/ml (case 2)) and the femoral vein blood (41.8 μg/ml (case 1), 72.1 μg/ml (case 2)). In vitreous humour, amphetamine concentration was also comparably low (case 1, 37.6 μg/ml). In case 2 the concentration of amphetamine in the vitreous humour was nearly twice as high (136 μg/ml) as in the femoral vein blood (72.1 μg/ml). It is well known that the concentration of a substance in the femoral vein blood may reflect the blood concentration at the time of death since post mortal changes appear to be negligible. However, it should be kept in mind that, if longer post-mortem time intervals occur, there could be minimal losses of substances through the thin wall of venous blood vessels. For this purpose, skeletal muscle is a good alternative, if no blood can be obtained during the autopsy. In our study we determined amphetamine concentrations of 68.9 μg/g and 92 μg/g in the skeletal muscle. The highest concentration of amphetamine was found in case 2 in the stomach content (14,000 μg/g), followed by the gall bladder (590 μg/g) and the brain (247 μg/g); in case 1 the highest amphetamine concentrations were found in urine with 2600 μg/ml and in the stomach content (1200 μg/g). The findings for the stomach content in case 2 leads to the assumption that the amphetamine absorption and distribution were not completed. Similar findings were made by Al-Asmari et al., who examined 235 cases of amphetamine-related death and found the highest amphetamine concentrations in stomach content (2.72 mg/l) and urine (6.3 mg/l) [27], even if the determined concentrations in stomach content were significantly lower than in our case (case 2). The concentrations of amphetamine in the brain (175 μg/g (case 1), 247 μg/g (case 2)), the liver (229 μg/g (case 1), 186 μg/g (case 2)), the myocardium (89 μg/g (case 1), 92 μg/g (case 2)) and kidney (128 μg/g (case 1), 95 μg/g (case 2)) were quite similar in both cases. A comparison of the two presented cases shows that both men survived the high dose of amphetamine only for a short time, since the concentrations of (R)- and (S)-amphetamine are nearly similar. In cases of longer survival time after ingestion of amphetamine, an increasing concentration difference between (R)- and (S)-amphetamine can be expected. To which extent the synthesis by-products (DPIA and 4-M-5-PP) in amphetamine preparations can provide an indication of the time of intake remains unclear and demands for further research.

## 6 Conclusion

The men in the two presented cases died after oral ingestion of greater amounts of liquid amphetamine preparations. The examination of the tissues and body fluids, obtained during autopsies, show that the highest amphetamine concentrations were found in the stomach, the urine and the gall bladder. Beside amphetamine one its synthesis by-products, DPIA, was detected in the heart blood (case 2). In cases of amphetamine intoxications, it should be proofed, if DPIA as well as the other synthesis by-products mentioned in this work could serve as markers for the time of amphetamine intake. The autopsy findings in case 2 imply that ingestion of highly concentrated amphetamine preparations may cause alterations, resembling those that are found after acid intake. If the injuries themselves do not serve as a cause of death (no organ ruptures or extensive bleeding), toxicological analysis should be performed.

## CRediT authorship contribution statement

**Evelyn Pawlik:** Writing – review & editing, Writing – original draft, Validation, Project administration, Methodology, Funding acquisition, Formal analysis. **Felix Mayer:** Writing – review & editing, Visualization, Methodology. **Oliver Temme:** Writing – review & editing.

## Ethical considerations

Not relevant.

## Declaration of competing interest

The authors declare that they have no known competing financial interests or personal relationships that could have appeared to influence the work reported in this paper.
